# Challenges in Pediatric Liver Retransplantation: A Technical Perspective

**DOI:** 10.3390/children11091079

**Published:** 2024-09-03

**Authors:** Carlotta Plessi, Roberto Tambucci, Raymond Reding, Xavier Stephenne, Isabelle Scheers, Giulia Jannone, Catherine de Magnée

**Affiliations:** 1 Pediatric Surgery and Transplantation Unit, Department of Surgery, Cliniques Universitaires Saint-Luc, ERN TransplantChild, ERN Rare Liver, Université Catholique de Louvain, 1200 Brussels, Belgium; 2 Pediatric Gastroenterology and Hepatology Unit, Department of Pediatrics, Cliniques Universitaires Saint-Luc, ERN TransplantChild, ERN Rare Liver, Université Catholique de Louvain, 1200 Brussels, Belgium

**Keywords:** pediatric liver transplantation, retransplantation, surgical technique, living-donor liver transplantation

## Abstract

Background/Objectives: Liver retransplantation (reLT) is the only option for pediatric patients experiencing graft loss. Despite recent advancements in surgical techniques and perioperative management, it remains a high-risk procedure. Our aim is to describe our experience in pediatric reLT, focusing on the technical aspects and surgical challenges. Methods: We systematically analyzed surgical reports from pediatric reLT performed at our center between 2006 and 2023 to identify recurrent intraoperative findings and specific surgical techniques. We focused on challenges encountered during different phases of reLT, including hepatectomy, vascular, and biliary reconstruction. Additionally, we compared patient and graft survival rates among different groups. Results: During the study period, 23 children underwent 25 reLT procedures at our center. Major surgical challenges included complex hepatectomy and vascular reconstructions, necessitating tailored approaches. Our analysis shows that patient and graft survival were significantly lower for reLT compared to primary transplantation (*p* = 0.002). Early reLT had a significantly lower graft survival compared to late reLT (*p* = 0.002), although patient survival was comparable (*p* = 0.278). Patient and graft survival rates were comparable between the first and second reLT (*p* = 0.300, *p* = 0.597). Patient survival tended to be higher after living-donor liver transplantation (LDLT) compared to deceased-donor liver transplantation (DDLT), although the difference was not statistically significant (*p* = 0.511). Conclusions: Pediatric reLT involves significant technical challenges and lower survival rates. Advances in perioperative management are crucial for improving outcomes. Further research is needed to optimize surgical strategies and evaluate the long-term benefits of LDLT in pediatric reLT.

## 1. Introduction

Liver retransplantation (reLT) is the only option for patients with irreversible graft loss. Classically, its rates in pediatric patients were reported to be between 9 and 29% [1]. However, in the last decades, reLT underwent a steady increase in prevalence, becoming the second most common indication for liver transplantation in children [2]. This trend can be interpreted in two ways: first, advances in surgical techniques and perioperative management have expanded the eligibility criteria for reLT; second, the ethical debate concerning the paucity of organs in the pediatric population has been attenuated by using split organs or living donors for the reLT. Despite these advancements, many studies have reported lower patient and graft survival rates after reLT compared to primary liver transplantation. As a matter of fact, reLT is a high-risk procedure because of the severity of the recipient’s illness and the technical challenges of the surgery [3]. In previous studies, reLT was categorized as early (within 30 days) or late (after 30 days) based on the delay from the primary transplantation. Each group shows specific risks and benefits, but no consensus has been reached on which timing presents the fewest technical challenges and offers the best survival outcomes [2,4]. Although many studies have compared the outcomes of living-donor liver transplantation (LDLT) and deceased-donor liver transplantation (DDLT), few have investigated this comparison in the context of reLT [5]. The primary aim of our study is to describe our experience in pediatric reLT, focusing on the technical aspects and surgical challenges. Our secondary aim is to perform a survival analysis across different patient subgroups.

## 2. Materials and Methods

### 2.1. Study Design

Following the previous work published by Bordeaux et al. [6], which focused on reLT at our center between 1984 and 2005, we have retrospectively reviewed the medical records of 23 consecutive pediatric patients (<18 years of age) who received reLT at the Cliniques Universitaires Saint Luc (Université Catholique de Louvain, Brussels, Belgium) between 1 January, 2006, and 31 December, 2023. The minimal post-reLT follow-up was six months.

### 2.2. Data Collection

Demographic and clinical data were collected from donor and recipient hospital records, as were data from the Eutotransplant registry (the international collaborative framework responsible for the allocation of donor organs in Austria, Belgium, Croatia, Germany, Hungary, Luxembourg, the Netherlands, and Slovenia).

Pre-retransplantation variables included data regarding primary transplantation (primary liver disease, age, anthropometric measures, PELD, and postoperative surgical complications) and retransplantation (the etiology of primary graft loss, age, anthropometric measures, and PELD).

Intraoperative parameters included the type of donor (deceased versus living donor), graft characteristics (type of graft, graft weight, and total ischemia time), immunological characteristics (ABO compatibility), and technical characteristics concerning venous, portal, arterial, and biliary reconstruction. Retransplantation was considered “early” if performed 30 days before the primary liver transplantation and “late” if performed after more than 30 days [6].

Post-retransplantation outcomes were finally analyzed, including surgical complications (venous, portal, arterial, and biliary), medical complications (acute or chronic rejection and post-transplant lymphoproliferative disorder), and graft and patient survivals.

### 2.3. Technical Aspects

Technical details regarding donor and recipient surgeries have been previously reported [6,7,8,9,10,11]. However, in our study, we systematically analyzed the surgical reports of all patients to identify recurrent intraoperative findings and surgical techniques specific to retransplantation surgery.

### 2.4. Post-Transplant Management and Immunosuppressive Protocols

General postoperative management has been previously described [8,9,12].

Regarding immunosuppression during primary liver transplantation, a steroid-free protocol has been used since 2001, combining tacrolimus and basiliximab [13].

By contrast, a steroid-free protocol is never used in reLT; in this case, tacrolimus is associated with steroids (methylprednisolone) at the dose of 2 mg/kg, with a standardized withdrawal scheme.

### 2.5. Data Analyses

Continuous variables were expressed as medians and ranges, and categorical variables were expressed as numbers and percentages. Kaplan–Meier curves were used to analyze patient and graft survival in different subgroups. It should be noted that, in the group of reLT, we considered the moment of the reLT as time zero and not the moment of primary liver transplantation. A chi-square test was performed to compare categorical variables between different groups.

### 2.6. Ethics Committee

The study was approved by the Hospital Institutional Review Board (reference 2023/04JUL/302, Belgian registration number B403).

## 3. Results

### 3.1. Study Population

**Figure 1:**
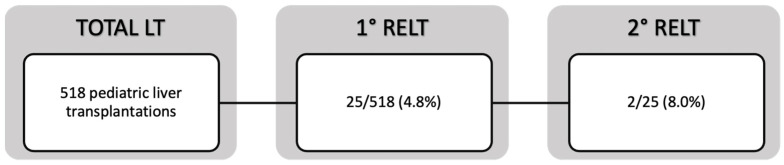
Flowchart representing our study population.

A total of 23 children, 12 males (52.2%) and 11 females (47.8%), underwent reLT during the 18-year-long study period: 21 patients (91.3%) were retransplanted once, while 2 patients (8.7%) received two reLT. This series represented 4.8% of a total of 518 pediatric liver transplantations performed during the same time interval at our institution. Among these, 414 (79.9%) were living-donor liver transplantations (LDLT), with 17 (4.1%) requiring retransplantation, and 104 (20.1%) were deceased-donor liver transplantation (DDLT), with 8 (7.7%) requiring reLT (*p* = 0.127). All told, 7 of 23 reLT patients (30.4%) underwent primary liver transplantation at a different institution. A flowchart representing our study population is shown in Figure 1.

Patients’ characteristics at primary liver transplantation are listed in Table 1.

Patients’ characteristics at reLT are shown in Table 2.

### 3.2. Technical Aspects of reLT

Both the hepatectomy phase and implantation phase were analyzed.

During dissection for graft removal, in 13 cases (52.0%), difficulties related to strong adhesions were described. In 12 cases (48.0%), hemorrhagic diathesis was reported in the operative protocol. In one case (4.0%), an intestinal perforation was detected during the dissection. In one case (4.0%), we experienced caval perforation with gas embolism, without hemodynamic consequences. No difference was found between early reLT and late reLT in terms of problems in the dissection phase (X2 = 0.37, *p* = 0.541).

During hepatic vein reconstruction, the first step was always the resection of the previous anastomosis to completely remove the tissue from the old graft. Then, in all cases, we performed a piggyback reconstruction. This was biangular in 10 cases (40.0%) and triangular in 15 cases (60.0%), changing according to the type of graft.

During portal reconstruction, in 21 cases (84.0%) a classic termino-terminal anastomosis was performed. In one case (4.0%), the portal vein of the graft was anastomosed to a venous jump graft already implanted in the superior mesenteric vein during a previous meso-rex shunt surgery performed due to portal vein thrombosis. In three cases (12.0%), the interposition of a venous graft between the graft portal vein and the splenomesenteric confluence of the recipient was necessary: in two cases, the reason for this was a complete thrombosis of the portal trunk; in one, case the portal vein had to be resected because of extreme fragility related to a post-necrotizing enterocolitis inflammatory environment. In one case (4.0%) due to a complete thrombosis of the portal trunk, including the spleno-mesenteric confluence, we interposed a venous graft between the graft portal vein and the superior mesenteric artery, with a terminal–lateral anastomosis. In all the cases, the vascular graft was an iliac graft derived from the same deceased donor.

During arterial reconstruction, in 16 cases (64.0%), a classic terminal–terminal anastomosis was performed. In seven cases (28.0%), a jump graft between the graft artery and the infrarenal aorta was necessary: in two cases, the reason for this was that the recipient artery was too thin; in five cases, it was required because the artery was completely thrombosed. The vascular graft used for the arterial reconstruction was, in four cases (16.0%), an iliac graft of the same cadaveric donor; in three cases (12.0%), it was an iliac graft from a previously deceased donor. In the latter case, the vascular graft had to be obtained no more than 48 h earlier to prevent endothelial damage [4]. In two cases (8.0%), the recipient artery was too short, necessitating the interposition of a gastroepiploic arterial graft from the living donor. Moreover, in three cases of cadaveric donation, an accidental lesion of an accessory hepatic artery of the graft was found: in one case, a simple re-anastomosis between the two stumps was sufficient; in the other two cases, the sectioned accessory artery was anastomosed using the gastroduodenal stump of the graft.

As regards the biliary system, in all cases, we performed a hepaticoenterostomy due to pre-existing Roux-en-Y bilio-digestive reconstruction. In 22 cases (88.0%), we performed a single biliary anastomosis; in 3 cases (12.0%), two anastomoses were necessary.

At the time of abdominal closure, the use of a temporary prosthesis was necessary in 5 cases (20.0%), while vacuum-assisted closure therapy (VAC) was used in only one case (4.0%). The technical aspects of reLT are summarized in Table 3.

### 3.3. Outcomes of reLT

Major complications (Clavien–Dindo III or more) occurred in 12 patients (52.0%) after reLT, and some of them underwent more than one procedure. We had an arterial complication (4.0%), a portal complication (4.0%), 3 (12.0%) anastomotic biliary complications, 2 (8.0%) non-anastomotic biliary complications, and 11 (44.0%) general complications (intra-abdominal bleeding, intestinal perforation, intestinal occlusion, enterocolitis, disseminated aspergillosis, …). In 12 cases, complications were managed with surgery, and in 3 cases they were managed via interventional radiology. Two patients (8.0%) underwent a second reLT, one due to a vascular complication and one due to a non-anastomotic biliary complication. A patient is currently on the waiting list for a second reLT for non-anastomotic biliary complications with recurrent cholangitis.

Regarding immunological complications, there were 7 cases (28.0%) with an acute rejection, 3 (12.0%) with a chronic rejection, and 2 (8.0%) with a post-transplant lymphoproliferative disorder (PTLD).

Three patients died during the same admission of the reLT: in one case, the cause of death was an abdominal compartment syndrome (patient 6 on post-reLT day 56); in one case, it was sepsis (patient 8 on postoperative day 10), in one case it was a massive gastric hemorrhage (patient 7 on postoperative day 10). All the others were discharged, with a median total hospital stay of 54.0 days (range 10.0–162.0 days) and a median stay in the intensive care unit of 7.0 days (range 1.0–89.0 days). Another patient died later, after discharge, for unexplained reasons (patient 2 on postoperative day 2005).

### 3.4. Patient and Graft Survivals

**Figure 2:**
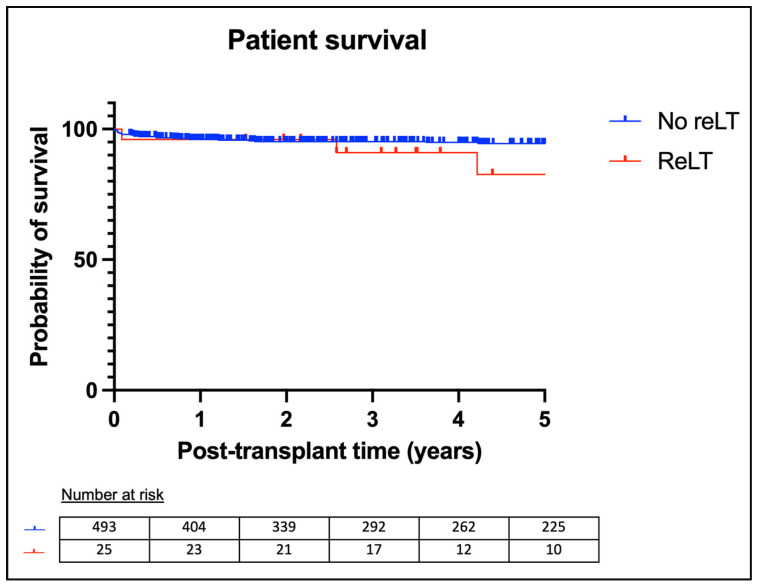
Kaplan–Meier patient survival analysis.

The 1-year patient survival rates were 98.0% for patients who did not require reLT (no-reLT group) and 96.0% for the reLT group. The 5-year patient survival rates were 94.5% in the no-reLT group and 82.7% in the reLT group. Kaplan–Meier analysis showed a significant difference in patient survival between the two groups (*p* = 0.02). Patient survival curves are shown in Figure 2.

**Figure 3:**
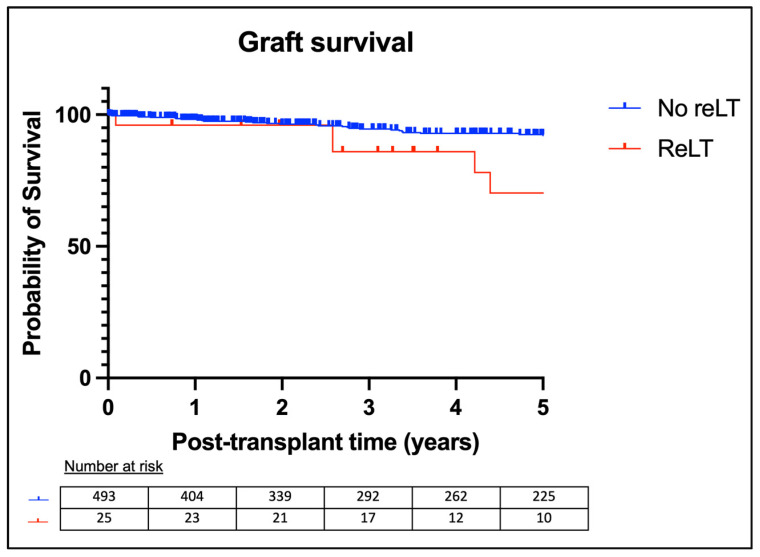
Kaplan–Meier graft survival analysis.

The 1-year graft survival rates were 98.2% for the no-reLT group and 96.0% for the reLT group. The 5-year graft survival rates were 92.5% for the no-reLT group and 70.3% for the reLT group. Kaplan–Meier analysis showed a significant difference in graft survival between the two groups (*p* = 0.02). Kaplan–Meier curves for graft survival are shown in Figure 3.

### 3.5. Living/Deceased Donor Comparison

Among the 25 studied cases, 8 reLT (32.0%) procedures took the graft from a living donor, and 17 (68.0%) took it from a deceased donor. The one- and five-year patient survival rates were 100% and 87.5%, respectively, in LDLT, versus 82.4% and 70.6% in DDLT. The one- and five-year graft survival rates were 100% and 58.3%, respectively, in LDLT, versus 76.5% and 65.5% in DDLT. Kaplan–Meier analysis did not show any significant differences in patient (*p* = 0.511) or graft survival (*p* = 0.194) between the two groups.

### 3.6. Early/Late reLT Comparison

Among the 25 studied cases, 3 reLT (12.0%) cases occurred between day 1 and day 30 post-LT (median day 29, range 12–30) versus 22 cases (88.0%) occurring beyond day 30 post-LT (median day 342, range 69–4490). One- and five-year patient survival rates were 66.7% in early reLT, versus 90.9% and 77.5%, respectively, in late reLT. One- and five-year graft survival rates were 33.3% in early reLT, versus 90.9% and 68.9%, respectively, in late reLT. Kaplan–Meier analysis showed a significant difference in graft survival between the two groups (*p* = 0.002), but no difference in patient survival (*p* = 0–278).

### 3.7. First/Second reLT Comparison

Among the 25 studied cases, 23 (92.0%) received their first reLT and 2 (8.0%) their second reLT. One- and five-year patient survival rates were 95.7% and 86.1%, respectively, in the first reLT, versus 100% and 50.0% in the second reLT. One- and five-year graft survival rates were 95.7% and 71.6%, respectively, in the first reLT, versus 100% and 50.0%, in the second reLT. Kaplan–Meier analysis did not show any significant differences in patient (*p* = 0.300) and graft survival (*p* = 0.597) between the two groups.

## 4. Discussion

The advancements in surgical techniques and peri-operative management have significantly improved survival rates among pediatric patients undergoing liver transplantation. Despite these improvements, a notable proportion of patients still face graft loss, leaving reLT as the only alternative to death. In pediatric patients, the ethical problem of reLT in the context of organ shortages is partially mitigated by using partial livers from living donors or split organs from deceased donors. Consequently, the global trend in transplant centers is to offer a second transplant to all patients with primary or secondary graft failure, except for those with absolute contraindications such as extrahepatic sources of sepsis, disseminated malignant tumors, and severe irreversible neurological injuries [4]. Over the past few decades, the number of pediatric reLT procedures has increased, becoming the second most common indication for liver transplantation. This underscores its growing importance in the field of transplant medicine. Survival outcomes have concurrently improved, as illustrated by Vock et al., who reported an 84.5% survival rate for reLT recipients between 2002 and 2018, compared to the 60% rate reported by Davis et al. between 1989 and 2006 [2].

Nevertheless, reLT remains a high-risk procedure, with survival rates still lower than those of primary liver transplantation [14]. In our series, patient and graft survivals were significantly lower for reLT patients compared to primary transplantation patients. Three out of four patients in our series died during the same admission without being discharged from the pediatric intensive care unit.

The nature of the risk differs between early and late retransplantation scenarios. In the instance of early retransplantation, patients typically present with severe illness, characterized by hemodynamic instability, coagulopathy, the necessity of urgent intervention, and meticulous perioperative management strategies. Conversely, late retransplantations are characterized by distinct challenges related to compromised clinical conditions exacerbated by chronic immunosuppression and technical complexities arising from factors such as strong vascular adhesion and distorted anatomy [2,14]. Some single-center studies reported poorer outcomes for children undergoing early reLT [4,15]. However, Vock et al. [2] found that early reLT resulted in better survival rates compared to late reLT, likely because the surgery was performed before major complications, like sepsis, could develop. In our series, graft survival was significantly lower for the early reLT group compared to the late reLT group, although there were no differences in 1-year patient survival. This data arises from the need for a third transplantation in two out of three patients in the early group. Moreover, Vock et al. [2] compared survival rates between patient subgroups based on the number of reLT, reporting that survival rates for second and third reLTs were significantly lower compared to first reLT and primary transplantation. Instead, a recent single-center study by Junger et al. [16] found comparable overall survival rates between first, second, and third reLT, despite the increased technical challenges. Likewise, we did not find in our series any difference in patient and graft survivals between the first and second reLT. In the comparison between living-donor liver transplantation (LDLT) and deceased-donor liver transplantation (DDLT), a recent systematic review and meta-analysis [5] showed that LDLT was superior in terms of patient and graft survival. Not many studies have compared reLT outcomes between living and deceased donors. Dai et al. [17] recently found that reLT using living-donor right-liver grafts can achieve excellent long-term survival rates in adults. Polat et al. [18] found higher survival rates in LDLT compared to DDLT in adult reLT patients, but the difference was not statistically significant. Similarly, our series showed that patient and graft survival rates tended to be higher with living donors in reLT, although the difference was not statistically significant, likely due to the small sample sizes.

Independently of the timing and the type of graft used, technical challenges in reLT are significant and varied. The hepatectomy phase is often marked by difficult adhesiolysis and consequential major bleeding. This phenomenon is likely attributable to transcapsular arterial neovascularization, a well-described occurrence associated with chronic failure after liver transplantation. It is characterized by the development of atypical collateral vessels systematically associated with intra-abdominal adhesions. As suggested by Herrmann et al. [19], a preemptive identification of this phenomenon through high-resolution preoperative ultrasound could facilitate the proper planning of the operation, predicting blood loss and indirectly the presence of extensive adhesions. A meticulous surgical technique is essential to avoid injury to other organs; in our experience, we encountered an intestinal lesion and a caval perforation during graft dissection. Despite our expectations, we did not find a statistically significant difference between the early reLT and the late reLT groups. However, this may be due to the small sample size in the early group, which included only three patients.

The implantation phase presents its own challenges, primarily due to the distortion of the vascular anatomy, often leading to the inadequacy of vessels required for vascular reconstruction. The reconstruction technique used in primary transplantation can influence reLT procedure, which requires a tailored approach. For example, piggyback anastomosis in the primary procedure may enhance the likelihood of preserving the cava during reLT, whereas side-to-side anastomosis often necessitates caval resection (CR) [20]. In our case series, we consistently avoided CR, likely due to the uniform use of the piggyback technique in primary transplants. In contrast with Laroche et al. [20], who always try to preserve the previous anastomosis with a patch of the previous graft’s vein, we prefer to remove all the tissue from the primary graft to decrease the risk of post-transplant Budd–Chiari syndrome.

Portal vein reconstruction can also present various challenges for the surgeons, each requiring tailored interventions. One scenario entails the presence of portal vein thrombosis, encountered in three of our patients. Early reLT may involve thrombectomy, but success depends on thrombosis not extending to the superior mesenteric vein [21]. For late reLT or extensive portal vein thrombosis, vascular bypass is typically required. Another scenario involves challenges related to the recipient’s portal vein status, such as compromised venous wall, diameter, and length. This was observed in one of our patients who presented with an extremely fragile portal vein in the context of necrotizing enterocolitis. Alternative options for reconstruction are possible, including iliac grafts from cadaveric donors, consistently used in our series, as well as autologous internal jugular veins or inferior mesenteric veins from living donors, particularly in the context of living-donor transplantation. In three of our cases, the venous graft was interposed between the portal branch of the graft and the splenomesenteric confluence, where a terminal–terminal anastomosis was performed. In one case, it was implanted on the superior mesenteric vein with a terminal–lateral anastomosis.

Arterial reconstruction poses similar challenges in the context of reLT, mirroring complexities observed in portal vein reconstruction. Under normal circumstances, terminal–terminal anastomosis between the recipient and graft artery is the preferred approach. However, complete thrombosis, the dissection of the recipient artery, or anatomical constraints may necessitate the use of arterial conduits. Reese et al. [22], in their meta-analysis, recognize reLT as a predicting factor regarding the need for arterial conduits, especially in the context of third transplantations. In our series, a notable proportion of reLT necessitated the use of arterial conduits, primarily because of arterial thrombosis or anatomical limitations such as the inadequate length or diameter of the recipient artery. Consistent with the existing literature [22], the vascular graft employed in most cases was an iliac graft from the same deceased donor. Consequently, the use of living donors has technical limitations, especially after arterial thrombosis. Another approach, as proposed by Martinez et al. [23], consists of utilizing a vascular homograft from a previous deceased donor, provided it has been preserved in a Ter solution for no longer than 48 h to mitigate endothelial damage. In reLT patients involving living donors, alternative arteries from the same living donor, such as the right gastroepiploic artery, have proven to be effective for arterial reconstruction [24,25,26]. In two of our cases, we interposed the gastroepiploic artery of the living donor between the recipient’s common hepatic artery and graft left hepatic artery. In all other cases, we performed an infrarenal aortic placement of the deceased iliac arterial conduit, which, according to the literature, represents the preferred inflow implantation site [22]. In our experience, in rescue circumstances, a pediculated recipient’s right gastroepiploic artery or splenic artery may be used for arterial reconstruction. An additional challenge we encountered in arterial reconstruction during liver retransplantation was the accidental injury of accessory hepatic arteries during the procurement or split phase [27]. Such an occurrence necessitates prompt intervention to re-establish continuity or implement alternative reconstruction strategies involving neighboring arteries. In our experience, we were able to reconstruct accidentally dissected accessory hepatic arteries on the gastroduodenal artery of the same graft. Failure to address these injuries can lead to compromised blood supply and subsequent necrosis of part of the graft.

Biliary reconstruction in pediatric reLT is typically less challenging due to the standardized use of Roux-en-Y hepaticojejunostomy in primary transplantation, as in our series. Thus, during reLT, biliary reconstruction is relatively straightforward, with the only viable option being an end-to-side anastomosis on the pre-existing loop. The study conducted by Sibuleski et al. [28] offers valuable insights, comparing different biliary reconstruction techniques during retransplantation in an adult population. Their findings indicated that there were no statistically significant differences between duct-to-duct anastomosis and Roux-en-Y hepaticoenterostomy in terms of postoperative outcomes.

Finally, staged abdominal wall closure may be necessary due to various factors such as graft–recipient weight ratio (GRWR) >2.5% (large for size), perioperative graft, bowel edema, or issues with graft positioning. This is particularly relevant in reLT with complex vascular reconstructions, where delayed abdominal closure after temporary prosthesis placement or vacuum-assisted closure therapy (VAC) may be preferred in order to manage excessive intra-abdominal pressure [29].

Several potential limitations should be considered when interpreting the results of this study. First, the monocentric and retrospective design of the study may limit its strength. Second, the small sample size and the limited number of early reLT in our series may affect the statistical power of our analyses.

## 5. Conclusions

In conclusion, the evolution of surgical techniques and peri-operative management has significantly improved survival rates among pediatric liver transplantation recipients. However, graft loss remains a challenge for a considerable portion of patients, necessitating reLT as the sole alternative to death. ReLT has emerged as the second most common indication for liver transplantation, reflecting its growing significance in transplant medicine. Despite these advancements, reLT remains a high-risk procedure, with survival rates that are inferior to those of primary liver transplantation.

Our series highlighted the difficulties of hepatectomy and vascular reconstruction during reLT. The nature of the risk differs between early and late retransplantation scenarios, with distinct challenges posed by each. Further research is needed to optimize surgical strategies and evaluate the long-term benefits of LDLT versus DDLT in pediatric reLT.

## Figures and Tables

**Table 1:** ReLT patients’ characteristics and data concerning primary liver transplantation (n = 23).

Primary Liver Disease	Biliary atresia	11 (47.8%)
	Cryptogenic cirrhosis	2 (8.7%)
	Hepatoblastoma	2 (8.7%)
	Metabolic disorders	2 (8.7%)
	Progressive familial intrahepatic cholestasis (type II)	2 (8.7%)
	Alpha-1 antitrypsin deficit	1 (4.3%)
	Crigler–Najjar syndrome	1 (4.3%)
	Hepatitis A	1 (4.3%)
	Overlap syndrome	1 (4.3%)
Age in Years [median (range)]		1.9 (0.6–14.0)
Weight in Kilograms [median (range)]		13.5 (7.0–41.0)
Z-score for Weight [median (range)]		−0.5 (−2.7–2.4)
Pediatric End-Stage Liver Disease (PELD) [median (range)]		5.4 (−10.0–30.0)
Urgency Code (According to Eurotransplant)		2 (8.7%)
Graft Type	Living donor	15 (65.2%)
	Deceased donor	8 (34.8%)
Graft Weight in Grams [median (range)]		270.0 (230.0–450.0)
Total Ischemia Time in Minutes [median (range)]		106.0 (58.0–552.0)
ABO-Incompatibility		3 (13.0%)
Surgical Complications	Arterial complications	5 (21.7%)
	Portal complications	4 (17.4%)
	Hepatic veins complications	3 (13.0%)
	Biliary complications	11 (47.8%)
	Other complications	5 (21.7%)

**Table 2:** ReLT patients’ characteristics and data concerning retransplantation (n = 25).

Etiology of Primary Graft loss	Rejection		13 (52.0%)
	Biliary complications		4 (16.0%)
	Arterial complications		3 (12.0%)
	Portal complications		3 (12.0%)
	Hepatic veins complications		1 (4.0%)
Age in Years [median (range)]			2.8 (0.7–14.1)
Weight in kg [median (range)]			13.5 (6.6–50.0)
Z-score for Weight [median (range)]			−0.9 (−3.4–0.3)
Pediatric End-Stage Liver Disease (PELD) [median (range)]			13.6 (−6.2–38.3)
Urgency Code (According to Eurotransplant)			13 (52.0%)
Timing	Early reLT		3 (12.0%)
	Late reLT		22 (88.0%)
Graft Type	Living donor		8 (32.0%)
		Left lateral lobe	4 (16.0%)
		Full left lobe	4 (16.0%)
	Deceased donor		17 (68.0%)
		Whole liver	4 (16.0%)
		Split liver	7 (28.0%)
		Reduced-size liver	6 (24.0%)
Graft Weight in Grams [median (range)]			355.5 (190.0–420.0)
Total Ischemia Time in Minutes [median (range)]			465.0 (80.0–960.0)
ABO-Incompatibility			1 (4.0%)
Surgical Complications	Biliary complications		5 (20.0%)
	Arterial complications		1 (4.0%)
	Portal complications		1 (4.0%)
	Hepatic veins complications		0 (0%)
	Other complications		10 (40.0%)

**Table 3:** Technical aspects of reLT.

N°	Graft	Hepatectomy	HV	PV	Artery	Biliary System	Abdominal Closure
1	DD	Adh + HD	BP	TT PVd-PVr	Iliac graft CHAd-IRA	HE	Primary
2	DD	Adh + HD + caval perforation	BP	TT LPVd-PVr	TT CHAr-CHAd	HE	Primary
3	DD	Adh + HD	BP	TT LPVd-PVr	Iliac graft LHAd-IRA	HE	Primary
4	LD	/	TP	TT LPVd-PVr	TT CHAr-LHAd	HE	Primary
5	DD	/	TP	TT LPVd-PVr	TT CHAr-CHAd	HE	Primary
6	DD	Adh + HD	TP	TT LPVd-PVr	TT CHAr-CHAd	HE	VAC
7	DD	/	BP	TT PVd-PVr	TT CHAr-CHAd	HE	Primary
8a	LD	Adh + HD	TP	TT LPVd-PVr	Iliac graft LHAd-IRA	HE	Prosthesis
8b	DD	HD	BP	TL PVd-SMR	Iliac graft CHAd-IRA	HE	Primary
9	DD	Adh + HD + intestinal perforation	BP	TT PVd-PVr	Iliac graft CHAd-IRA	HE	Primary
10	DD	Adh	BP	TT PVd-PVr	TT CHAr-CHAd + ALHAd-GDAd	HE	Primary
11	DD	Adh + HD	TP	TT LPVd-PVr	TT CHAr-CHAd	HE	Primary
12a	LD	/	TP	TT LPVd-PVr	TT CHAr-LHAd	HE	Primary
12b	DD	Adh	BP	TT PVd-PVr	TT CHAr-CHAd	HE	Primary
13	LD	/	TP	TT LPVd-PVr	Gastroepiploic graft LHAd-CHAr	HE	Prosthesis
14	DD	HD	TP	TT PVd-PVr	Iliac graft CHAd-IRA	HE	Primary
15	LD	/	TP	TT LPVd-PVr	TT CHAr-LHAd	HE	Primary
16	LD	/	TP	TT LPVd-PVr	TT RHAr-LHAd	HE	Primary
17	DD	Adh	TP	Iliac graft LPVd-SMC	Iliac graft LHAd-IRA	HE	Prosthesis
18	LD	Adh	TP	Iliac graft LPVd-SMC	Gastroepiploic graft LHAd-CHAr	HE	Primary
19	DD	HD	BP	Iliac graft LPVd-SMV	TT CHAr-CTd + ALHAd-GDAd	HE	Primary
20	DD	/	TP	Iliac graft LPVd-SMC	TT CHAr-ALHAd	HE	Prosthesis
21	DD	Adh + HD	BP	TT PVd-PVr	TT CHAr-CHAd	HE	Primary
22	LD	/	TP	TT LPVd-PVr	TT CHAr-LHAd	HE	Primary
23	DD	Adh + HD	TP	TT LPVd-PVr	TT CHAr-CHAd	HE	Primary

HV: hepatic vein; PV: portal vein; DD: deceased donor; LD: living donor; Adh: adhesions; HD: haemorragic diatesis; BP: biangular piggyback; TP: triangular piggyback; TT: termino-terminal; TL: termino-lateral; PVd: donor portal vein; PVr: recipient portal vein; LPVd: donor left portal vein; SMR: shunt mesorex; SMC: splenomesenteric confluence; SMV: superior mesenteric vein; CHAd: donor common hepatic artery; CHAr: recipient common hepatic artery; IRA: infrarenal aorta; LHAd: donor left hepatic artery; ALHAd: donor accessory left hepatic artery; GDAd: donor gastroduodenal artery; RHAr: recipient right hepatic artery; CTd: donor celiac trunk; HE: hepaticoenterostomy.

## Data Availability

The data presented in this study are available on request from the corresponding author.
